# Broad-Spectrum Antibiotic or G-CSF as Potential Countermeasures for Impaired Control of Bacterial Infection Associated with an SPE Exposure during Spaceflight

**DOI:** 10.1371/journal.pone.0120126

**Published:** 2015-03-20

**Authors:** Minghong Li, Veronica Holmes, Houping Ni, Jenine K. Sanzari, Ana L. Romero-Weaver, Liyong Lin, Alejandro Carabe-Fernandez, Eric S. Diffenderfer, Ann R. Kennedy, Drew Weissman

**Affiliations:** 1 Division of Infectious Diseases, Department of Medicine, Perelman School of Medicine, University of Pennsylvania, 3610 Hamilton Walk, Philadelphia, PA 19104, United States of America; 2 Department of Radiation Oncology, Perelman School of Medicine, University of Pennsylvania, 3610 Hamilton Walk, Philadelphia, PA 19104, United States of America; Albert Einstein College of Medicine, UNITED STATES

## Abstract

A major risk for astronauts during prolonged space flight is infection as a result of the combined effects of microgravity, situational and confinement stress, alterations in food intake, altered circadian rhythm, and radiation that can significantly impair the immune system and the body’s defense systems. We previously reported a massive increase in morbidity with a decrease in the ability to control a bacterial challenge when mice were maintained under hindlimb suspension (HS) conditions and exposed to solar particle event (SPE)-like radiation. HS and SPE-like radiation treatment alone resulted in a borderline significant increase in morbidity. Therefore, development and testing of countermeasures that can be used during extended space missions in the setting of exposure to SPE radiation becomes a serious need. In the present study, we investigated the efficacy of enrofloxacin (an orally bioavailable antibiotic) and Granulocyte colony stimulating factor (G-CSF) (Neulasta) on enhancing resistance to *Pseudomonas aeruginosa* infection in mice subjected to HS and SPE-like radiation. The results revealed that treatment with enrofloxacin or G-CSF enhanced bacterial clearance and significantly decreased morbidity and mortality in challenged mice exposed to suspension and radiation. These results establish that antibiotics, such as enrofloxacin, and G-CSF could be effective countermeasures to decrease the risk of bacterial infections after exposure to SPE radiation during extended space flight, thereby reducing both the risk to the crew and the danger of mission failure.

## Introduction

The potential major risk of developing infections due to impaired immune function during prolonged space flight has been a continued concern since the beginning of human spaceflight. Potential sources of organisms that cause infection include both endogenous organisms, such as latent viruses (Varicella zoster virus and Epstein Barr virus) that become reactivated during space flight and commensals, as well as, exogenous organisms that are present in the spacecraft or other astronauts [1–7]. High incidences of bacterial infections have been documented during and soon after spaceflight [4,6,8,9]. Approximately 50% of Apollo crew members contracted bacterial or viral infections, which included gastroenteritis; respiratory, skin, urinary tract, and fungal infections and flu-like illness (reviewed in [10]). *Pseudomonas aeruginosa* has been identified as a pathogenic organism shown to cause infections during spaceflight [1] and it has been used in animal models of spaceflight to understand the reduced ability to clear infections [8,11].

A large literature exists on the impairment of the immune system by spaceflight and model systems that potentially lead to a reduced ability to control a variety of infections (reviewed in [9,12,13]). Space factors shown to potentially impair immune function include microgravity [14–17], radiation [7], physiological stress [18,19] disruption of circadian rhythms [20,21] and altered nutrition [22]. The alterations in immune function that have been documented during space travel in most studies include a decrease in NK cell number and function, a reduction in peripheral T-cell counts, altered cytokine production [23,24], and modified granulocyte number and function [25,26]. Thus, a major concern of a dysregulated immune system in the closed environment of a spacecraft is the altered ability to control bacterial, fungal, viral, and parasitic invasions [5,7,27] and the loss of immunosurveillance leading to tumor growth [28]. In a previous study, we observed that hindlimb suspension (HS) and solar particle event (SPE)-like radiation at least additively impaired the ability to control a bacterial challenge and compromised the granulocyte response [11]. In this study, we hypothesized that two countermeasures with completely different modes of action could control *a* bacterial infection in mice subjected to the HS model of microgravity plus exposure to SPE-like radiation. The first countermeasure used was a broad-spectrum orally available antibiotic (enrofloxacin), and the second countermeasure used was granulocyte colony-stimulating factor (G-CSF, Neulasta). Both were found to be highly effective in preventing morbidity and aiding the clearance of systemic bacteria.

## Materials and Methods

### Humane care and use of animals

This study was carried out in strict accordance with the recommendations in the Guide for the Care and Use of Laboratory Animals of the National Institutes of Health. The protocol was approved by the Institutional Animal Care and Use Committee (Assurance # A3079–01) of the University of Pennsylvania. Facilities housing the animals involved were accredited by the Association for Assessment and Accreditation of Laboratory Animal Care, International (AAALAC) and inspected regularly by the U.S. Department of Agriculture (USDA).

### Animals

Female C3H/HeNCr MTV- mice, 6 weeks of age and weighing 18–22 g, were purchased from the NCI-Frederick National Laboratory. Animals were housed with controlled temperature and light cycle, in standard laboratory vivarium caging (prior to experimentation) with *ad libitum* access to both food and water. Experimental procedures commenced after a one-week acclimation. Humane conditions were employed during all aspects of the studies. During bacterial challenges, animals were monitored at least once per day using the morbidity scoring in Table 1. If an animal increased by 3 points or remained 2 points elevated for greater than 24 hrs, they were considered a study endpoint and removed and euthanized. Mice were euthanized by CO_2_ inhalation, where a gradual fill rate of 10–30% chamber volume per minute displacement was ensured using a regulated fill valve. After cessation of respiration, euthanasia was ensured by cervical dislocation. No procedures were employed that required the use of analgesic or anesthetic pretreatment.

**Table 1:** Assessing morbidity.

Score	Morbidity level	Characteristics
1	No indication of morbidity	Normal, well groomed, alert, active, good condition, asleep or calm, normal appetite
2	Mild morbidity	Not well groomed, awkward gait, slightly hunched, mildly agitated
3	Moderate morbidity	Rough hair coat, squinted eyes, moves slowly, walks hunched and/or slowly, depressed or moderately agitated, slight dehydration, pruritic, restless, uncomfortable, not eating or drinking
4	Severe morbidity	Very rough hair coat, eyes sunken (severe dehydration), slow to move or nonresponsive when coaxed, hunched, dyspnea, self-mutilating, violent reaction to stimuli or when approached

### Hindlimb Suspended (HS)

Mice were maintained under HS conditions, as described previously [29]. The HS mice were prepared for suspension by placing a Steri-Strip (3M) at the base of the tail. An adjustable bead chain was placed parallel to the tail and taped with athletic tape. Individual mice were suspended by the tail at 30° head-down tilt with no load bearing on the hindlimbs. The adjustable bead chain with an end coupling was allowed to freely move across a rod at the top the cage. Access to food and water was ensured using both water bottles and gel packs and food distributed around the floor of the cage. Animals demonstrated no serious adverse effects or pronounced weight loss. Experimental animals were singly housed in custom-built cages whether normally loaded or subjected to HS.

### Experimental procedure and groups

**Fig 1:**
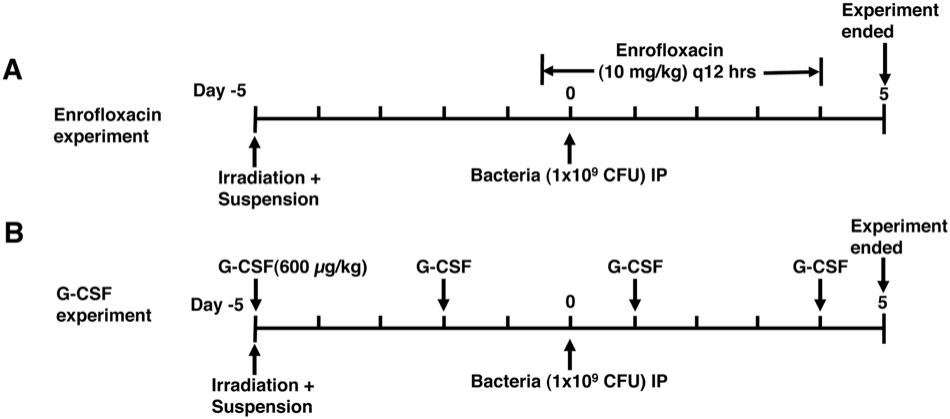
Diagram of experimental procedure. Groups of 10 mice per treatment condition were irradiated or sham with 2 Gy of proton or gamma radiation and then hindlimb suspended or not. (A) Mice began treatment with enrofloxacin (10 mg/kg) subcutaneously 12 hrs before bacterial challenge and were dosed every 12 hrs. (B) Mice began treatment with G-CSF (600 μg/kg) subcutaneously after irradiation and suspension and were dosed every 3 days. Five days post-irradiation, mice were challenged with *Pseudomonas aeruginosa* (10^9^ CFUs, IP). Mice were followed for 5 days and blood was obtained for quantitation of bacterial CFUs and granulocytes.

Animals were randomly assigned to experimental groups. Groups of 10 mice per treatment per experiment were used due to HS cage limitations. Mice were irradiated and then placed in HS. Immediately after irradiation, G-CSF (Neulasta, Amgen) treatment was started at 600 μg/kg every 3 days. For the enrofloxacin studies, animals were treated or not with enrofloxacin (Bayer Animal Health) five days after irradiation, 10 mg/kg subcutaneously every 12 hrs starting 12 hrs before bacteria injection (Fig. 1). Mice were challenged with bacteria 5 days after irradiation/suspension. Blood was collected by cheek lancet on day 5-post bacteria challenge and analyzed for CFUs and granulocytes. All blood draws and intraperitoneal bacteria inoculations were performed without anesthesia with animals maintained in the HS apparatus.

The following experimental groups were used: 1) Control + PBS: singly housed and unirradiated mice, receiving subcutaneously injected PBS, and infected with *P*. *aeruginosa*. 2) Control + treatment (enrofloxacin or G-CSF): receiving subcutaneously injected enrofloxacin or G-CSF and infected with *P*. *aeruginosa*. 3) Radiation + suspension (R+S) + PBS: HS and irradiated mice receiving subcutaneously injected PBS and infected with *P*. *aeruginosa*. 4) R + S + treatment (enrofloxacin or G-CSF): receiving subcutaneously injected enrofloxacin or G-CSF and infected with *P*. *aeruginosa*.

### Radiation

The mice were constrained in custom designed Plexiglas chambers with dimensions of 7.2 cm × 4.1 cm × 4.1 cm and whole body irradiated with gamma or proton radiation at a dose of 2 Gy, delivered in a single fraction at a 0.5 Gy/min dose rate. All mice, irradiated and sham-irradiated, were maintained in the same conditions and period of time corresponding to those treated with 2 Gy of irradiation. Gamma radiation was delivered using a Cesium 137 Gammacell 40 irradiator (Nordion). The mice were exposed to SPE-like proton radiation using an IBA cyclotron (IBA Particle Therapy) in the Roberts Proton Therapy Center of the University of Pennsylvania. The 230 MeV proton beam derived from the cyclotron was reduced using the energy selection system to a nominal energy of 151 MeV or range of 16 cm water equivalent thickness (WET). The reduced beam was delivered in double scattering mode with a spread out Bragg peak (SOBP) modulation width of 5 cm. A 23 cm × 17 cm opening in the tungsten multi-leaf collimator shaped the beam to a useable field size (>95% of uniform dose within the flat region) of 20.6 cm × 17 cm at the gantry isocenter. The mouse enclosures were arranged so that they formed a 16.4 cm × 14.2 cm target area. The center of the enclosure array was placed at the gantry isocenter with an additional 11 cm WET of Solidwater (Gammex, Inc.) placed directly in front of the enclosure array, further reducing the proton beam energy to approximately 74 MeV or range of ~4.5 cm WET. Five centimeters of Solidwater were placed directly behind the enclosure array. The mouse enclosures were irradiated with a range of proton energies forming the uniformly modulated dose region of the SOBP. The dose averaged linear energy transfer (LET) of the proton radiation is low (10 keV/μm) within the mid-SOBP where the mice are located and rises to higher LET (> 10 keV/μm) towards the downstream edge of the SOBP, which lies beyond the mouse enclosures [30]. Dosimetry verification was performed before the irradiations with a 2D ion chamber array (I’m*RT* MatriXX, IBA dosimetry) placed at a depth of 13.3 cm WET. These irradiation conditions result in a homogeneous dose distribution of SPE-like proton irradiation in the mice. Mouse proton irradiations at the Roberts Proton Therapy Center have been described previously [11,31–33].

### Enrofloxacin treatment

Mice in the antibiotic treated groups received 10 mg/kg enrofloxacin starting 12 hrs before the bacterial infection procedure (Fig. 1A) and then twice a day (every 12 hrs) until the end of the experiment. Mice were injected subcutaneously in the back of the neck with a total volume of 100 μl, and antibiotic untreated groups were injected with 100 μl of PBS on the same time schedule for comparison.

### G-CSF treatment

G-CSF (Neulasta) was kindly provided by Amgen, Inc. The duration and dosing of G-CSF were established by Romero-Weaver [31]. Mice in the G-CSF treatment group received 600 μg/kg injected subcutaneously in the back of the neck in a total volume of 100 μl per animal, at a 3 day interval that started after irradiation (Fig. 1B). G-CSF untreated groups were injected with 100 μl of PBS.

### Bacterial infection

**Fig 2:**
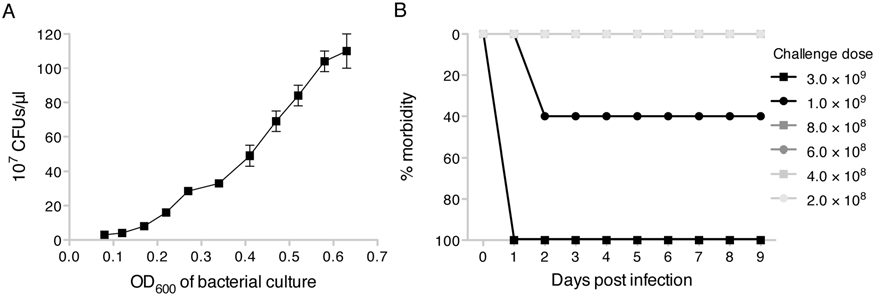
Determination of bacterial challenge dose. (A) The association between the OD_600_ of the bacterial culture and the number of CFUs/μl was determined for *Pseudomonas aeruginosa* by plating dilutions of bacterial culture and quantitating colonies. The OD_600_ quantitation of CFU/μl was then used to determine the numbers of CFUs in the bacterial culture in real time. (B) The amount of bacteria that untreated C3H/HeN mice could control was determined by challenging with increasing amounts of CFUs intraperitoneally and following for morbidity as defined in Table 1.

A systemic infection model was used with modification of previously described methods [8,11,34]. *Pseudomonas aeruginosa* (Schroeter) Migula (ATCC, 27853) frozen stock was inoculated on tryptic soy agar (TSA) plates for colony isolation. After overnight incubation at 37°C, one colony was transferred to 20 mls of fresh tryptic soy broth (TSB), followed by shaking at 220 rpm at 37°C. Growth curves were prepared by plotting the optical density (OD) at 600 nm (WPA CO 800 Cell Density Meter, Biochrom) versus calculated bacterial counts determined by plating (Fig. 2A) to determine the challenge inoculum. The culture was stopped when an OD_600_ of 0.20–0.23 was reached (corresponding to a bacterial concentration of approximately 2 × 10^11^). The bacterial cultures were diluted to 10^9^ CFUs/ml. The challenge inoculum was spread on TSA plates to confirm the challenge dose. For infection, 1 ml of diluted bacterial suspension was inoculated intraperitoneally (IP) into mice. Initial studies in untreated control mice determined the relationship between the amount of bacteria delivered and morbidity (Fig. 2B).

Blood was obtained 5 days after bacterial inoculation for determination of blood CFUs and granulocyte counts. Changes in the number of animals that did not clear bacteria or experienced an increase in morbidity, as defined in Table 1, were calculated.

### CFU determination

Tenfold serial dilutions of each blood sample were made in tubes containing 0.9 ml of PBS prior to plating 0.1 ml from selected dilutions onto TSA plates. Plates were incubated for 24 hrs at 37°C and individual colonies were counted to determine the number of CFUs.

### Granulocyte counting

Whole blood (100 μl) was stained for Ly-6G (neutrophils, DCs) (clone 1A8, BD Bioscience), CD14 (monocytes) (clone rmC5–3, BD Bioscience), and F4–80 (monocytes) (clone BM8, Biolegend) to quantitate granulocytes. AccuCount Fluorescent Particles (12.5 μl of 5.2 μm size, Spherotech, Inc.) were added to calculate absolute counts (cells/μl). Whole blood was lysed after staining with FACS lysis buffer (BD Bioscience) and analyzed on a FACS Canto A (BD Bioscience) with DiVa Software and further analyzed using FlowJo Analysis Software (Tree Star Inc.). Cells were gated for forward and side scatter and dead cells (a very small fraction) were excluded. The total numbers of granulocytes were quantified as: (the number of events (Ly-6G high, CD14-, and F4–80-) divided by (number of events for the AccuCount particles) times (number of AccuCount particles per 12.5 μL times volume of test sample used (0.1 ml)). In some samples, absolute counts of granulocytes were measured using volumetric/flow-rate calibration [35,36]. Stained and lysed whole blood was analyzed for a fixed amount of time that resulted in identical amounts of volume analyzed. All samples from each experiment were analyzed at the same time to avoid possible variation in flow rates that could occur at different days, temperatures, or relative humidities.

### Statistics

The results from groups of 10 mice were averaged and comparisons were analyzed by one-way ANOVA using Bonferroni’s correction for multiple comparisons (Prism 5.0). Morbidity scores (Table 1) were calculated daily and animals were considered morbid if their score increased by 3 points or remained 2 points elevated for 24 hrs or death of the animal. Statistical significance between groups for Kaplan-Meier analyses was measured using the Mantel-Cox log rank test.

## Results

Mice were subjected to radiation (2 Gy of gamma or proton) and then placed in HS for 5 days prior to bacterial challenge [11] (Fig. 1). As CFUs cannot be calculated in real time, since it requires overnight culture for accurate measurement, the OD of the bacterial culture at 600 nm was used and correlated with CFUs/μl (Fig. 2A) [11], which was used as a less accurate surrogate for the challenge dose. A dose of bacteria that resulted in 20–40% morbidity in non-suspended and non-irradiated control mice was used (Fig. 2B). To further decrease the variation in the number of CFUs delivered, challenges were performed using bacterial cultures within a narrow OD_600_ range of 0.20–0.23. *P*. *aeruginosa* was delivered intraperitoneally. The dose used was higher than the dose used in a previous study [11], thereby increasing the difficulty to obtain protection, although similar protection was observed when lower doses of bacteria that could be controlled in untreated mice were used. Mice were followed for 5 days after bacterial challenge (Fig. 1). Mice received 10 mg/kg of enrofloxacin q12 hr (Fig. 1A) or 600 μg/kg of G-CSF q3 days (Fig. 1B), injected subcutaneously in the back of the neck in a total volume of 100 μl; enrofloxacin or G-CSF untreated groups were injected with 100 μl of PBS for comparison. Blood was obtained on day 5 post bacterial challenge and plated on agar in serial dilutions to quantitate the number of CFUs present and signs of morbidity (Table 1) were followed daily throughout the experiment. Animals were considered at the experimental endpoint when morbidity increased 3 points or an increase of 2 points was present for more than 24 hrs, using the criteria outlined in Table 1. Non-suspended and non-irradiated mice were placed in radiation enclosures for the same amount of time and non-suspended mice were individually caged, similar to HS mice. The relative increase in morbidity or lack of clearance of bacteria due to HS and radiation was consistent across experiments. Gamma radiation was employed as a reference radiation and to estimate relative biological effectiveness (RBE). Two Gy of gamma radiation gave similar results as 2 Gy of SPE-like proton irradiation, suggesting an RBE = 1, although we did not have dose response analyses to definitely determine RBE.

**Fig 3:**
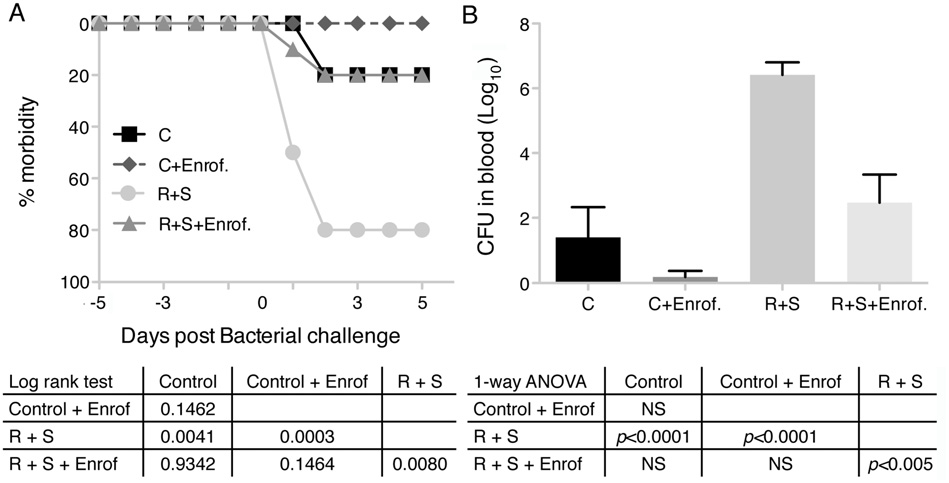
Treatment with enrofloxacin reduces morbidity and increases bacterial clearance in irradiated and hindlimb suspension mice systemically challenged with *Pseudomonas aeruginosa*. Groups of 10 mice per treatment group were irradiated with 2 Gy of gamma rays and hindlimb suspended or not and injected subcutaneously with either PBS or enrofloxacin (10 mg/kg) every 12 hrs starting 12 hrs before bacterial challenge. All groups were exposed to *Pseudomonas aeruginosa* by intraperitoneal injection and followed. Morbidity scores (Table 1) were calculated daily (A) and animals were considered morbid if their score increased by 3 points or remained 2 points elevated for more than 24 hrs. C = control, R + S = irradiated + hindlimb suspended. (B) Bacteremia was quantitated 5 days after challenge by diluting and plating blood followed by colony counting. Values are means ± SEM of log_10_ of colony-forming units (CFU). One-way ANOVA with Bonferroni’s correction for multiple comparisons was used to test statistical significance of changes in CFUs in blood. Statistical significance of Kaplan-Meier curves was measured by Mantel-Cox log rank test.

Morbidity was significantly reduced in enrofloxacin treated HS and gamma irradiated mice (*p* = 0.0080, Fig. 3A). The bacterial load was also significantly decreased in the antibiotic-treated group compared with the PBS treated group (Fig. 3B). Control mice receiving antibiotic showed a trend toward reduced morbidity compared to control mice receiving PBS. Similar reductions were found when the bacterial load was analyzed. Mice subjected to proton radiation and hindlimb suspension had complete protection from bacterial challenge with treatment by enrofloxacin (data not shown).

**Fig 4:**
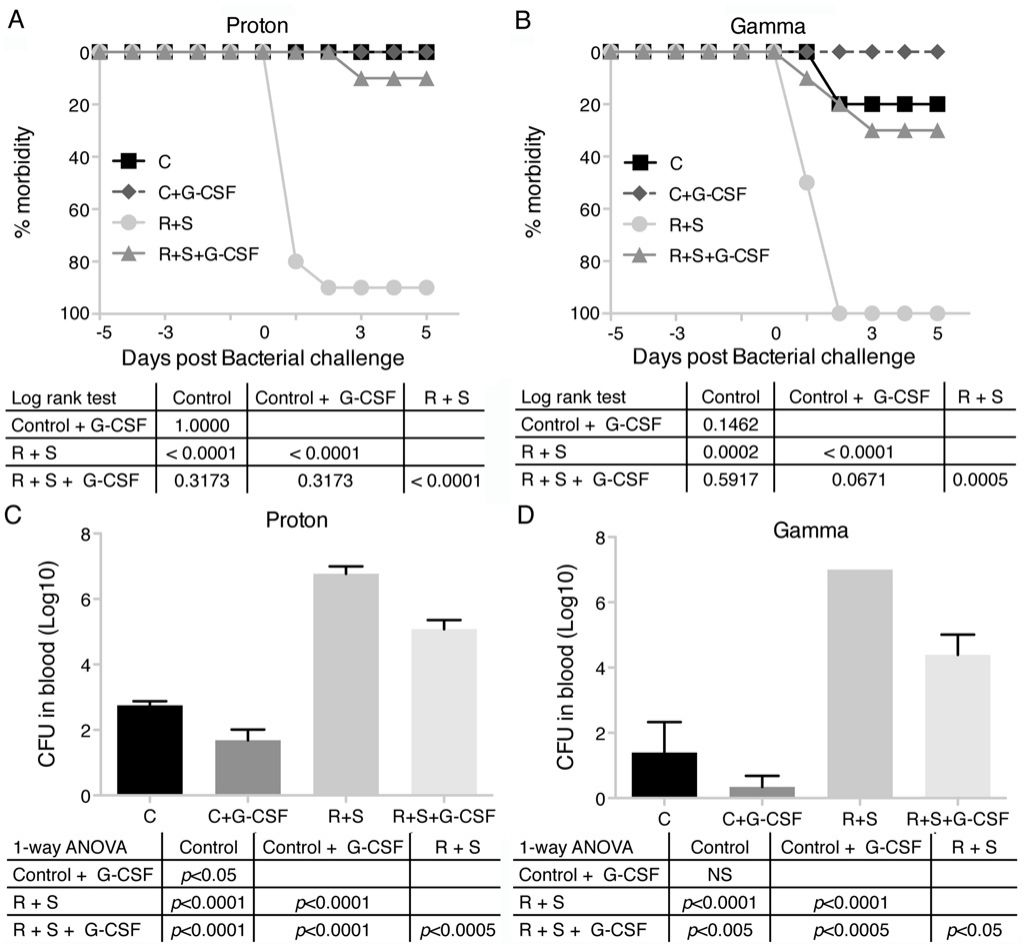
Treatment with G-CSF reduces morbidity and increases bacterial clearance of irradiated and hindlimb suspension mice systemically challenged with *Pseudomonas aeruginosa*. Sets of 10 mice per treatment group were irradiated with 2 Gy of protons or gamma rays and hindlimb suspended or not and injected with PBS or G-CSF (600 μg/kg) by the subcutaneous route every 3 days until the end of the experiment. All groups were exposed to *Pseudomonas aeruginosa* by intraperitoneal injection 5 days after irradiation and followed. Morbidity scores (Table 1) were calculated (A—proton and B—gamma rays) daily and animals were considered morbid if their score increased by 3 points or remained 2 points elevated for 24 hrs. C = control, R + S = irradiated + hindlimb suspended. Bacteria in blood were quantitated 5 days after infection (C—proton and D—gamma rays). Values are means ± SEM of log_10_ colony-forming units (CFUs). One-way ANOVA with Bonferroni’s correction for multiple comparisons was used to test the statistical significance of changes in CFUs. Kaplan-Meier curve statistical significance was measured by Mantel-Cox log rank test.

Antibiotic treatment is associated with the development of antibiotic resistant bacteria, especially in a closed environment [37], such as a spacecraft. An alternative countermeasure that would avoid the pressure to develop antibiotic resistance was assessed. To evaluate the ability of G-CSF (Neulasta) to enhance the resistance to infection with *P*. *aeruginosa*, G-CSF was subcutaneously injected into control and hindlimb suspended and irradiated mice prior to bacterial challenge. In discussions with NASA flight surgeons, it was determined that a treatment with a growth factor, such as G-CSF or erythropoietin, would likely be used immediately or soon after an exposure to an SPE containing a significant amount of radiation. Thus, we chose to begin G-CSF treatment immediately after radiation exposure and continued treatment until the end of the experiment. In both proton and gamma ray irradiated animals, G-CSF treatment resulted in a dramatically decreased bacterial load and morbidity compared with mice receiving PBS (Fig. 4). A significant decrease in morbidity in the hindlimb suspended and proton irradiated G-CSF treated group (R + S + G-CSF) compared to the suspended and irradiated PBS treated group (R + S, *p* < 0.0001, Fig. 4A) was observed. Similar results were obtained using gamma rays (*p* = 0.0005, Fig. 4B). For neither type of irradiation did the suspended and irradiated plus G-CSF treated groups return to the level of morbidity found in the control mice, suggesting effective but incomplete protection. The bacterial load was significantly decreased in the G-CSF group compared with the PBS group (Fig. 4C and D), but again, the levels of bacteria in the blood of R + S + G-CSF treated mice did not drop to that observed in the control treated mice.

**Fig 5:**
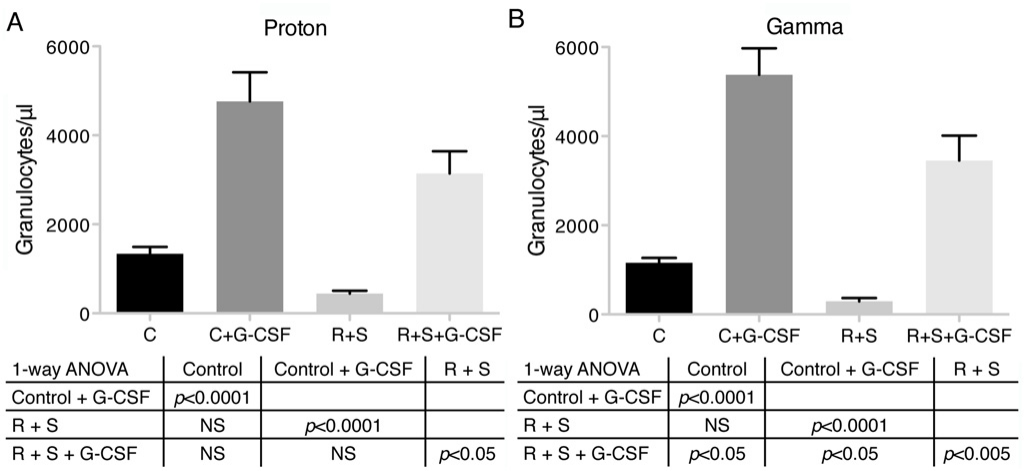
Effect of G-CSF on peripheral blood granulocyte counts during systemic bacterial challenge of irradiated and hindlimb suspended mice. Sets of 10 mice per treatment group were irradiated with 2 Gy of protons or gamma rays and hindlimb suspended or not and injected with PBS or G-CSF (600 μg/kg) every 3 days. All groups were exposed to *Pseudomonas aeruginosa* by intraperitoneal injection and followed. After 5 days, the number of granulocytes, Ly-6G high, CD14-, and F4–80- in peripheral blood was calculated using AccuCount fluorescent particles. (A) proton treated and (B) gamma ray treated. C = control, R + S = irradiated + hindlimb suspended. Values are means ± SEM. One-way ANOVA with Bonferroni’s correction for multiple comparisons was used to test statistical significance.

Peripheral blood granulocyte counts were determined in the G-CSF experiments. A significant reduction of granulocytes 10 days after irradiation in the R + S group was observed (Fig. 5A and B). The groups that received G-CSF had significantly elevated granulocyte counts that were associated with protection from infection. Similar results were observed in the proton and gamma radiation experiments.

## Discussion

An astronaut can receive up to 2 Gy of proton radiation to the bone marrow during a strong SPE while on an extravehicular excursion [38,39], which is a dose that has been shown to suppress the bone marrow and alter immune function [40–42]. An optimal countermeasure would be avoidance of exposure to an SPE, but they cannot be predicted and it is believed that an astronaut may have little (less than one hour) or no notice of an incoming SPE. A second potential countermeasure would use shielding, but the cost per kilogram of transporting necessary materials is prohibitive. We adapted models of space travel and SPE-radiation to analyze potential countermeasures that could be useful in the setting of SPE exposure during extended space travel. We used the standard HS model of microgravity (reviewed in [43,44])) that includes situational and confinement stress, fluid shifts, altered diet intake, altered circadian rhythms, and non-load bearing status. In these studies, a dose of 2 Gy of either SPE-like proton or gamma radiation, as a standard radiation source, was used. Two mechanistically non-overlapping countermeasures were analyzed. The first used a single broad-spectrum orally available antibiotic and the second used the cytokine granulocyte colony stimulating factor. Both significantly, and in the case of the antibiotic, completely, avoided systemic infection and morbidity, following challenge with *Pseudomonas aeruginosa*, a pathogenic bacteria that has caused infections in astronauts [1].

Hindlimb suspension, as a model of the stresses endured by an astronaut during space travel, has been demonstrated to impair the granulocyte response [11], including a reduction in the ability of phagocytes to kill bacteria and generate superoxide anion radicals [45]. Previous studies have also observed a range of immune defects induced by this model, including thymic involution similar to that observed with space flight [46], reductions in T cell activation and cytokine production, and multiple impairments to immune effector function [43,47–49] Identified mechanisms include an increase in stress hormone corticosteroid levels [45]. Those stress hormones (i.e., glucocorticoids) inhibit the migration of neutrophils and macrophages into inflammatory sites [25,50] and alter acquired immune cell functions [51,52]. Radiation in the 2 Gy range results in a significant decrease in granulocyte counts in peripheral blood [11,31,32] and other organs [40,53,54] and multiple impairments to immune function have been reported (reviewed in [13,55,56]). Combining the HS model of spaceflight with SPE-like radiation results in a greatly impaired ability to control bacterial infections in inbred and outbred strains of mice challenged with bacteria by systemic and inhalation routes [11].

A number of countermeasures have been investigated to ameliorate the consequences of reduced immune function during spaceflight conditions. Antioxidants, such as *N*-acetycysteine, ascorbic acid, α-lipoic acid, L-selenomethionine, coenzyme Q10, and vitamin E succinate have been used (reviewed in [13]), and applied to human cell lines and mice subjected to SPE-like radiation [57,58]. Supplemental dietary nucleotides, uracil/uridine and pyrimidines, have been demonstrated to possess immunoprotective effects and enhance immune function [59]. An active hexose correlated compound (AHCC) has been demonstrated to have a positive effect on the immune system of humans [60] and was tested on reducing infectious complications in hindlimb suspended mice [61–63]. Photosensitization, bacteriophage, and green tea extract therapy have been studied to reduce or prevent bacterial infection [64–66]. The anti-*Pseudomonas aeruginosa* antibody panobacumab combined with antibiotic co-treatment of murine pneumonia with antibiotic resistant *P*. *aeruginosa* has been reported [67]. The disadvantage of this approach for space travel is the need to identify the organism before treatment and the storage of many different bacteria specific antibodies.

The results described in the present study indicate that antibiotic and G-CSF treatment can prevent and reduce *P*. *aeruginosa* infection in mice exposed to HS and SPE-like proton or gamma radiation, respectively. The timing after exposure to an SPE was determined based on discussions with NASA flight surgeons. The initiation of therapy after a large SPE was chosen to provide the greatest protection to the astronauts, whereas, a patient who develops neutropenia after radiation therapy does not start antibiotic therapy until a fever or obvious signs of infection are noted. The application of this paradigm to astronauts, who may be weeks to months away from Earth, could endanger the mission and potentially their lives. It is anticipated that a countermeasure would be employed for an astronaut after a certain dose of SPE radiation is received and continued for a predetermined length of time, in the absence of symptoms of infection. Expansion to a wider range of antibiotics and other organisms, including unidentified bacteria, as would be expected during space travel, is easily justified, as long as the bacteria causing the infection is sensitive to the chosen antibiotic and granulocytes are involved in protection.

Antibiotic treatment is a rapid and efficient method to treat bacterial infections and is effective in our model system of space travel and SPE radiation exposure, but multiple investigations have observed that greater concentrations of antibiotics were generally required to inhibit microbial growth in space compared to ground-based controls; this suggests that increased microbial proliferation, altered virulence and decreased antibiotic efficiency may be present [68,69]. In addition, the development of bacterial resistance could lead to difficulties with subsequent antibiotic use and will likely further alter the gut microbiota of astronauts, the significance of which is currently unknown. The potential difficulties associated with the development of antibiotic resistance are well known on Earth and additional complications could occur for astronauts on extended missions, which temper the use of antibiotics, especially multiple doses, during explorer type missions. Thus, it is possible that the efficacy of antibiotics may be compromised and the potential adverse effects may be expanded during space missions and significant further studies are needed to understand the potential antagonistic effects of antibiotic use during extended space travel, which will have to be considered in the choices of countermeasures to be employed.

We demonstrate the effectiveness of 2 different countermeasures to reduce the risks of infection following exposure to the highest doses of SPE-like proton radiation that astronauts could receive during extended space travel. An orally available broad-spectrum antibiotic was completely effective, even when irradiated mice were challenged with a dose of bacteria that resulted in morbidity in control mice. The main disadvantages of antibiotic use are the risks associated with not identifying the causative organism(s) for the infection, the potential for a resistant bacterium to develop and the likelihood of developing resistance with repeated use of an antibiotic. These can be addressed by employing multiple different classes of antibiotics and potentially systems that can identify bacteria and antibiotic resistance that have been used on the international space station [70]. G-CSF, although not completely effective in reducing morbidity in our study to the level of control challenged mice, was highly effective. Its mechanism of activity does not involve, and is not hampered by, antibiotic resistance, which makes it a useful alternative countermeasure. Co-treatment with antibiotic and G-CSF may offer a promising countermeasure for protection against bacterial infection in space flight, especially after exposure to significant doses of SPE radiation. Our data further demonstrate the risk of immune dysfunction during extended space travel outside of the protection of the Earth’s magnetic field and identifies 2 different countermeasures that effectively reduce or curtail the risk of infection and associated morbidity.
